# Overview of dendritic cells and related pathways in autoimmune uveitis

**DOI:** 10.1515/biol-2022-0887

**Published:** 2024-09-09

**Authors:** Fan Zhao, Jing-Sheng Yu

**Affiliations:** Graduate School of Hunan University of Traditional Chinese Medicine, Changsha, 410000, Hunan, China; Ophthalmology, The First Affiliated Hospital of Hunan University of Traditional Chinese Medicine, Pharmaceutical University, Changsha, 410007, Hunan, China

**Keywords:** autoimmune uveitis, dendritic cell, T cells, signaling pathway, review

## Abstract

Dendritic cells (DCs) play a crucial role in bridging innate and adaptive immune responses. They are widely distributed in various tissues and organs, including the eyes. In the ocular context, permanent DCs are present at the peripheral edge of the retina and the peripapillary area in an immature state. However, during the inflammatory process, DCs become activated and contribute to the development of uveitis. This review focuses on introducing the characteristics and status of DC-induced uveitis, exploring factors that can influence the status of DCs, and discussing feasible methods for treating DCs in both experimental autoimmune uveitis animal models and humans. It emphasizes the importance of further research on molecular pathways and signaling pathways that regulate the function of DCs. For example, investigating molecules such as cytotoxic T-lymphocyte-associated protein 4, which inhibits the B7-CD28 co-stimulatory interaction, can help improve immune homeostasis. The aim is to identify new therapeutic targets and develop targeted strategies for DCs, such as DC vaccine therapy or the use of immune modulators. These approaches can be tailored to the immune characteristics and disease manifestations of individual patients, enabling personalized treatment strategies. This may include the personalized design and precise medication of DC therapy, with the ultimate goal of improving treatment efficacy while minimizing adverse reactions.

## 1 Introduction

Uveitis, an autoimmune-related eye disease, is a principal contributor to blindness [1]. It is featured by recurring retinal and uveal inflammation, with an incidence of about 75–714 cases per 100,000 people [2,3]. Indeed, young adults, especially those around the age of 35, are susceptible to uveitis, which is characterized by a spectrum of clinical manifestations, including varying degrees of reduced vision, dry eyes and tears, pain, photophobia, ciliary muscle congestion, anterior chamber empyema, vitreous opacity, and post-corneal deposits [4,5]. Uveitis cases are commonly classified into infectious uveitis and non-infectious uveitis (NIU) in clinical practice. It is widely recognized that NIU is associated with autoimmune or immune-mediated mechanisms [6]. Moreover, research has provided evidence that genetic factors and susceptibility to uveitis are linked to the involvement of T cells and B cells in the immune response, signal pathways, and environmental factors [7,8]. For example, increasing the level of miRNA-223-3p can inhibit the expression of NLRP3 inflammasomes, thereby inhibiting inflammation-related signaling pathways, reducing M1 macrophage polarisation levels, and increasing M2 macrophage polarisation levels, thereby exerting a regulatory effect on macrophage polarisation balance in uveitis rats; miRNA-30b-5p can downregulate the expression of Notch pathway-related molecules such as Notch1 and Dll4, thereby inhibiting Notch pathway activation and achieving therapeutic effects on uveitis [9]; the imbalance between autoreactive pathogenic effector T cells, including helper T cells 1 (Th1) and Th17 lymphocytes, and regulatory T cells (Tregs), is responsible for the pathogenesis of autoimmune uveitis [9]. In addition, conventional drugs for the treatment of autoimmune uveitis are mostly glucocorticoids, immunosuppressants, biological agents, non-steroidal anti-inflammatory agents, and cycloplegic agents [10,11]. The pathogenesis of uveitis remains poorly understood, and there is still much uncertainty surrounding it.

## 2 Methods

To ensure a transparent and rigorous review process, a systematic approach was employed for the selection of relevant original research papers. The following steps were undertaken.

### 2.1 Identification of databases

Multiple academic databases were searched to capture a comprehensive range of relevant studies. The databases included:

- Chinese Biomedical Journal Database;
- Chinese Hospital Digital Library (CHKD);
- MEDLINE Database; and
- PubMed.

### 2.2 Search strategy and keywords

A comprehensive search strategy was developed to identify relevant original research papers for this review. The primary keyword used was “uveitis.” The search strategy was tailored to each database, including the Chinese Biomedical Journal Database, CHKD, MEDLINE Database, and PubMed, to optimize the retrieval of relevant studies. The search terms employed included “autoimmune uveitis,” “dendritic cells,” “dendritic cell activation,” “dendritic cell function,” “dendritic cell therapy,” “immune response,” “signaling pathway,” “molecular pathways,” “immune modulators,” “dendritic cell vaccine,” “T cells,” and “review.” These keywords and search terms were combined using Boolean operators to refine the search and ensure the inclusion of original research papers that contribute to the understanding of dendritic cells (DCs) in the context of autoimmune uveitis.

### 2.3 Inclusion and exclusion criteria

Clear inclusion and exclusion criteria were established to guide the selection of studies. The criteria included: (1) studies published in peer-reviewed journals; (2) original research papers that investigated the role of DCs in autoimmune uveitis; (3) studies available in English or Chinese language; and (4) studies published up to the date of the literature search.

### 2.4 Systematic retrieval

The systematic retrieval of studies was conducted according to the predefined search strategy and inclusion/exclusion criteria. The search was performed independently by two reviewers to minimize bias and ensure consistency.

### 2.5 Quality assessment

The quality and relevance of the included studies were assessed to evaluate their scientific rigor and validity. Any discrepancies or disagreements between the reviewers were resolved through discussion and consensus.

## 3 DCs

DCs, which were first isolated from monocytes by Steinman and Coh in 1973, are necessary members of the body to initiate autoimmunity [12]. DCs are so named because of their dendritic or pseudopodia-like protrusions observed during their maturation process [13]. DCs are not only considered the most potent antigen-presenting cells (APCs) in mammals but also have the ability to activate naive T cells. They efficiently capture, process, and present antigens, playing a critical role in the immune response [14]. Langerhans cells are originally derived from macrophage progeny, with a high functional similarity to DC cells. Mature DCs are characterized by high expression of major histocompatibility complex II (MHC-II) molecules, which are crucial for antigen presentation, as well as co-stimulatory factors such as CD80, CD86, and others [15]. These molecules enable DCs to initiate the differentiation of naive T cells into various subsets, including Th1, Th2, Th17, and Treg cells. Moreover, DCs have the ability to migrate to ocular tissues and stimulate the secretion of various cytokines [15]. In addition to their role in initiating immune responses, DCs play a vital role in sustaining immune responses by efficiently capturing antigens and presenting them to T cells following processing [16]. These reports, taken together, highlight the indispensable role that DCs play in autoimmune uveitis disease.

**Figure 1:**
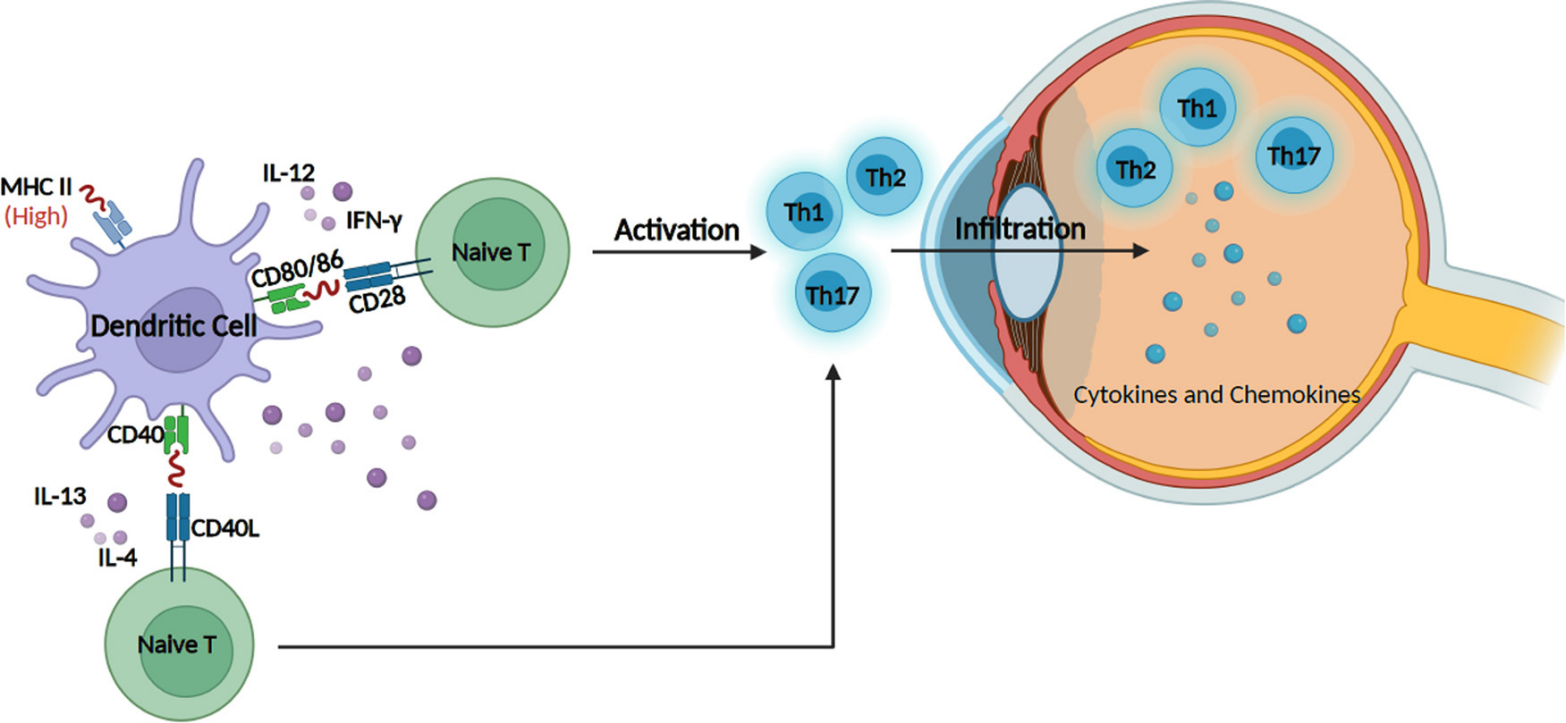
The pathogenesis of autoimmune uveitis.

Experimental autoimmune uveoretinitis (EAU) is a well-established animal model widely used in uveitis research [14,17]. Table 1 provides an overview of the roles played by different types of DCs in autoimmune uveitis. Previous studies have highlighted the critical involvement of DCs in mediating autoimmune uveitis. Specifically, it has been observed that rats injected intravenously with mature DCs exhibited more severe uveitis compared to the control group. Conversely, rats injected with immature DCs experienced milder uveitis compared to the control group [18]. Furthermore, Suzuki et al. demonstrated that inhibiting DC maturation using 5-aminoimidazole-4-carboxamide-1-beta-d-ribofuranoside effectively alleviated EAU inflammation. This finding suggests a close relationship between DC maturation, the occurrence of EAU, and the severity of inflammation. It further supports the notion that DCs are intricately involved in the onset and progression of autoimmune uveitis [18,19]. In addition, DCs are capable to activate T cells, secreting various cytokines, induce and promote the differentiation of activated T cells, which is implicated in regulating Th differentiation [20]. Among them, DCs secrete Th1 cytokines such as interleukin (IL)-12 and interferon (IFN)-γ, which induce T cells to differentiate toward Th1 [21]. On the other hand, DCs can also generate Th2-type cytokines such as IL-4 and IL-13, induce T cells to shift toward Th2, and cause allergic diseases [22]. During the pathogenesis of autoimmune uveitis, precursor cells of DCs originating from the bone marrow migrate to peripheral blood vessels. In the presence of antigens and other substances in the body’s microenvironment, these precursor cells differentiate into immature DCs. Upon stimulation by cytokines and chemokines, immature DCs migrate to the eye tissues where they recognize, process, and present antigens. Subsequently, they undergo maturation into mature DCs. Mature DCs exhibit high expression of MHC-II molecules and co-stimulatory factors involved in antigen presentation, such as CD80 and CD86. These mature DCs then migrate to the draining lymph nodes. In the lymph nodes, they secrete a series of cytokines that induce the differentiation of naive T cells into various T-cell subsets, including Th1, Th2, Th17, and Treg cells. Subsequently, the above-mentioned activated T cells migrate to the eye tissues and cause uveitis through the secretion of cytokines [15], suggesting that the differentiation and maturation of DC are involved in the occurrence of uveitis. What is noteworthy is that the core role of DC in EAU disease is to present antigens to CD4^+^ T cells, which eventually activate and cause an immune response. In EAU disease, retinal antigen-specific CD4^+^ T cells can induce inflammation. McMenamin et al. report that there are APCs in the iris and ciliary body of normal rats, which can obtain antigens on both sides of the blood–eye barrier, and then activate the antigens to specifically act on T cells [23]. More importantly, Dando et al. believe that DCs may be local APCs in the induction of uveitis [24]. In addition, another study reports that the regulation of IL-10 can inhibit the development of EAU inflammation and can significantly reduce the proliferation of interphotoreceptor retinoid-binding protein-specific T cells and the production of IFN-γ [25]. It can be concluded, accordingly, that DC is directly related to the occurrence of autoimmune uveitis and the severity of inflammation, and many cytokines regulate uveitis diseases through complex network functions (Figure 1).

**Table 1:** Role of different types of dendritic cells in autoimmune uveitis

Dendritic cell types	Primary function	Expression in autoimmune uveitis	Related signaling pathways
Plasmacytoid DC (pDC)	Secretion of IFN-α and IFN-β	Activation and accumulation during inflammation	TLR7/9-MyD88-IRF7
Conventional DC (cDC)	Secretion of IL-12, IL-6, and TNF-α	Mainly activates and regulates CD4^+^ and CD8^+^ T cells	TLR4-MyD88/TRIF-NFκB
Monocyte-derived DC (moDC)	Secretion of TNF-α, IL-1β, IL-6, and IL-12	Mainly activates CD4^+^ T cells and regulates inflammation and immune response	TLR2/4/8-MyD88-NFκB
Langerhans Cells (LC)	Antigen presentation, maintaining local immune homeostasis, secretion of IL-1, IL-6, IL-10, and TNF-α	Maintain immune homeostasis mainly by presenting antigens and activating local T cells (including CD4^+^ T cells, CD8^+^ T cells, and regulating T cells)	TLR2/4/6-MyD88-NFκB

### 3.1 Inducing DC activation mechanisms in infectious and NIU

The mechanisms underlying DC activation differ between infectious and NIU. In the case of infectious uveitis, the potential mechanism by which infectious agents induce immune responses and contribute to uveitis involves the binding of pathogen-associated pattern molecules on the surface of pathogenic microorganisms to corresponding NOD-like receptors. This interaction triggers DC activation and functional alterations that facilitate the differentiation of Th1 and Th17 cells. Consequently, the production of specific cytokines is promoted or inhibited, ultimately leading to the onset of uveitis [22].

In NIU, due to the disruption of the blood–ocular barrier, exposure to autoantigens, or invasion of exogenous antigens, DCs are activated and present their own or foreign antigens to Th cells, triggering an autoimmune response; in experimental endogenous and autoimmune uveitis, an increase in intracellular DCs is an important factor leading to uveitis. The increase of DCs in the uveal tissue may promote the expression of inducible nitric oxide synthase, synthesize NO, participate in the onset and development of disease, and regulate immune cell infiltration [19].

### 3.2 The mechanism of action of DCs in uveitis

Immature DCs are distributed in various ocular tissues including the cornea (epithelial and stromal layers), iris stroma, ciliary epithelium, suprachoroidal space, adjacent blood vessels, neural tissues penetrating the sclera, and retina. These immature DCs possess a strong capacity for antigen uptake and processing but exhibit weak antigen presentation ability. They can stimulate mixed lymphocyte responses *in vitro* and have the ability to induce immune tolerance. Immature DCs secrete cytokines such as IL-10 and TGF-β, which contribute to immune regulation. In the vitreous humor, the ocular microenvironment promotes immune tolerance and maintains immune balance. Immature DCs express low levels of MHC molecules and show little to no expression of co-stimulatory molecules like CD80, CD86, as well as adhesion molecules such as CD40, CD44, and CD54 [16].

During inflammation, immature DCs undergo maturation, or mature DCs migrate to the site of ocular lesions. The capacity of mature DCs to absorb antigens is significantly reduced compared to immature DCs. However, their ability to present antigens is greatly enhanced, surpassing that of B cells and macrophages by several hundred times. Mature DCs exhibit a robust capacity to promote immunological activation and can trigger mixed lymphocyte responses [15]. Mature DCs secrete various cytokines, including TNF-α, IFN-α, IL-12, IL-15, IL-21, IL-26, IL-28, and others. These cytokines contribute to the generation of inflammation, activation of adaptive immunity, and control of the differentiation of Th0 cells into Th1 or Th2 subsets. Mature DCs express high concentrations of MHC molecules, co-stimulatory molecules, adhesion molecules, and integrin molecules (such as β1 and β2). They also possess distinctive markers such as CD1a, CD11e, and CD83 [4].

## 4 Costimulatory molecules that activate T cells during DC initiation of immune response

### 4.1 B7 molecules

When DC initiates an immune response, the activation of T cells requires two signals: one is that TCR recognizes the antigen peptide-MHC complex indicated by APC and the other is the interaction of co-stimulatory molecules between T cells and APC. B7 and CD28 are a pair of important co-stimulatory molecules. The binding of B7 family ligand molecules to the CD28 receptor can not only provide co-stimulatory signals that induce T cell activation but also may generate co-inhibitory signals [26]. The B7 molecules belong to the members of the immunoglobulin (Ig) superfamily, including B7-1 (CD80), B7-2 (CD86), B7-H1 (PD-L1), B7-DC (PD-L2), B7-H2 (ICOSI), B7-H3 (CD276), B7-H4 (VTCN1), and B7-H6 [27]. Freeman cloned the cDNAs of B7-1 and B7-2 for the first time and performed sequence analysis. B7-1 (CD80) and B7-2 (CD86) are members of co-stimulatory signal molecules that have been identified, which are distributed on the surface of DCs and natural killer (NK) cells. Among them, B7-1 is mainly expressed in lymphocytes, such as DC and monocytes, and is inductively expressed in B cells. B7-1 can bind to two different receptors on T cells, CD28 and cytotoxic T-lymphocyte-associated protein 4 (CTLA-4), and provide a positive signal that activates T cells and a negative signal that inhibits T-cell responses. B7-2 is expressed on APC. In the immune response, B7-2 binds to the receptor CD28, and the receptor is concentrated on the immune synapse, leading to the activation and proliferation of T cells. Importantly, T-cell activation mainly depends on B7-2 [28]. As co-stimulatory molecules in the immune process, CD80 and CD86 can initially act on CD4^+^ T lymphocytes. Among them, CD80 can induce helper T cells to differentiate into Th1 cells, and CD86 can induce Th cells to differentiate into Th2 cells [29]. The co-stimulatory molecules CD80 and CD86 of mature DCs each play an important role in the immune response, which can cause a series of cytokines to promote the occurrence of uveitis [30].

### 4.2 CD28

CD28 family molecules belong to the Ig superfamily, which are type I transmembrane proteins [31]. It can be divided into positive co-stimulatory molecules that activate T cells and negative co-stimulatory molecules that induce T-cell tolerance according to different functions [32,33]. For example, positive co-stimulatory molecules include CD28 and ICOS, and negative co-stimulatory molecules include CTLA-4, PD-1, and NKp30 [34,35]. Among them, CD28 is a type I transmembrane glycoprotein receptor widely distributed on the surface of T cells [36]. CD28 binds to the two ligands B7-1 and B7-2 expressed on APC to provide an important positive co-stimulatory signal for the initial activation of T cells [37]. In patients with autoimmune diseases, there is an elevation in total IgE levels and/or IgE autoantibodies. In the case of autoimmune uveitis, IgE autoantibodies specific to retinal S antigen have been detected. Normally, IgE is the Ig with the lowest concentration in human serum. Its production involves the stimulation of B cells by IL-4 and IL-13, followed by interactions with B-T cells on the cell surface to complete antibody class switching. Upon recognition of foreign antigens, IgE initiates a signaling cascade through high-affinity FcεR I and low-affinity FcεR II FcεR receptors. This signaling cascade ultimately leads to mast cell and eosinophil degranulation, as well as the activation of Th2 responses.

### 4.3 CTLA-4 coinhibits B7-CD28 to regulate immune homeostasis and protect tissue integrity

Consequently, the release of biogenic amines, lipid mediators, proteases, and cytokines occurs, which can trigger allergic reactions in affected individuals. In addition, CTLA-4 is a leukocyte differentiation antigen, a transmembrane receptor on T cells, and it shares the B7 molecular ligand with CD28. The combination of CTLA-4 and B7 molecules, furthermore, induces T-cell anergy and participates in the negative regulation of immune response. This co-suppression plays an important role in autoimmune diseases, transplant rejection, tumors, and infectious diseases [38]. Studies have found that CTLA-4 is capable of attenuating the response of activated T cells and facilitating the inhibitory function of regulatory Treg [39].

**Figure 2:**
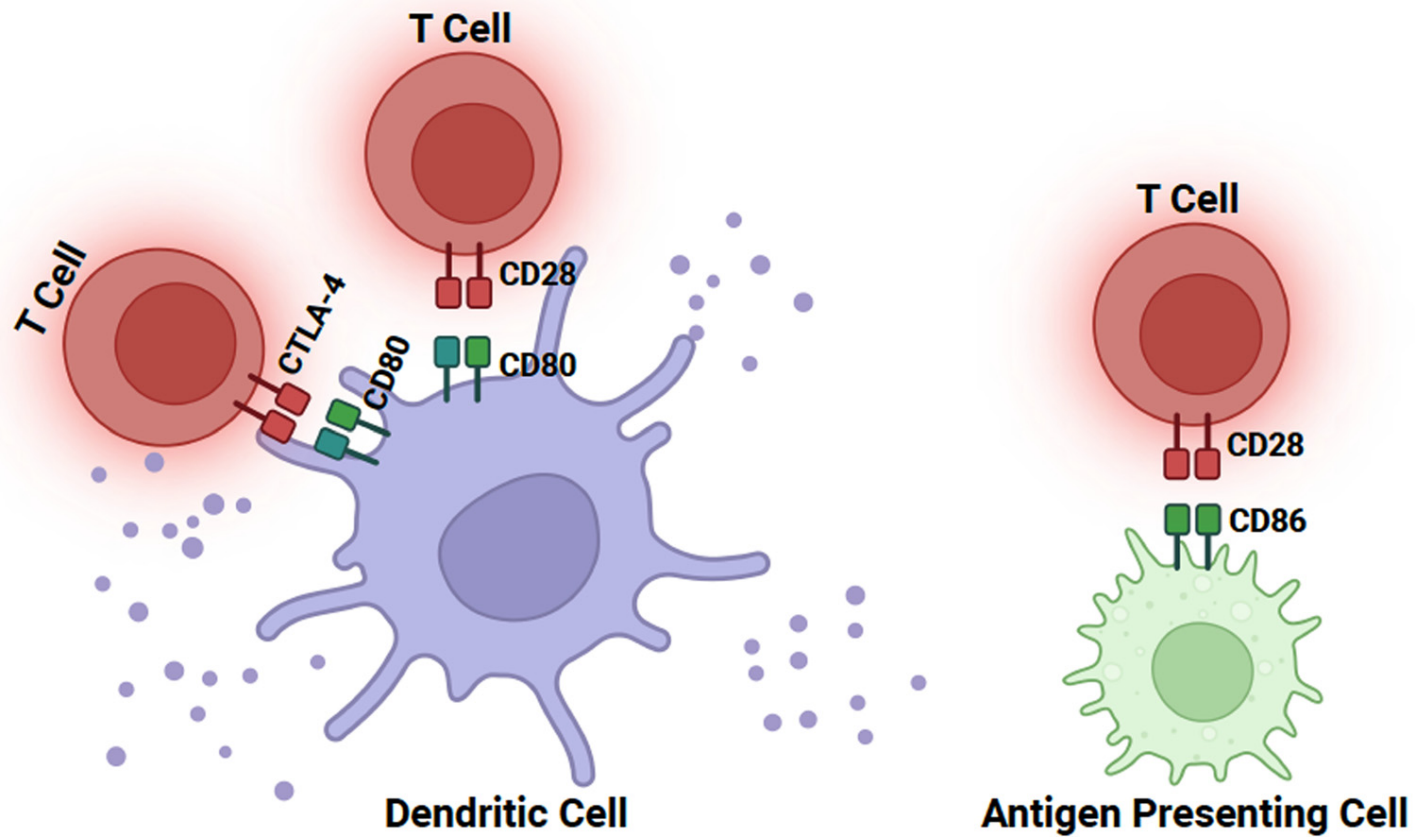
T cells interact with CD80/86 on DC and APC via CTLA-4 and CD28.

On the one hand, CD80 and CD86 have dual functions in regulating T-cell immune responses. First, they can inhibit the proliferation of T cells by interacting with CTLA-4, leading to T-cell apoptosis (as shown in Figure 2). Additionally, CD80 and CD86 negatively modulate the overall T-cell immune response by reducing the stimulatory activity mediated by T-cell receptor and CD28 [40,41]. On the other hand, CD80 and CD86 can enhance the transcription of IL-2 and increase IL-2 receptor expression by binding to CD28 [42,43]. The expression level of IL-2 on T cells drives the proliferation of T cells and can also prevent T cells from apoptosis by fostering the expression of recombinant human B-cell lymphoma factor 2 [44]. Excessive co-stimulation of CD80 and CD86 can lead to the activation of autoreactive T cells, which are implicated in the development of autoimmune diseases [45]. T cells play a crucial role in immune responses, and their activation and inhibition are tightly regulated by regulatory proteins such as CD28 and CTLA-4 [46]. The activity of these proteins is modulated by a pair of ligands known as CD80 and CD86, which can bind to their receptors non-covalently [47]. CD28 plays a significant role in enhancing T-cell activation. It can inhibit T-cell activation when CTLA-4 binds to its ligands [48]. CD28 may also exert inhibitory signals or compete with CD28 for the B7 receptor, thereby reducing T-cell activation. In the presence of CD28, the inhibitory effect of CTLA-4 on CD8 T cells is enhanced. Due to the inhibitory function of CTLA-4, targeting CTLA-4 has shown promise as a therapeutic approach in animal models of autoimmune diseases and transplant rejection. Blocking CTLA-4 and B7 interactions can stimulate T-cell immune responses. For example, CTLA-4Fc can bind to B7, thereby interrupting the B7-CD28/CTLA-4 pathway and reducing the intensity and frequency of inflammation in the anterior chamber in EAU. From another perspective, CTLA-4 knockout mice experience severe autoimmune diseases and eventually die due to excessive lymphocyte proliferation within 3–5 weeks. Therefore, the proper immune response in autoimmune uveitis relies on maintaining a delicate balance between CD28-mediated T-cell activation and CTLA-4-mediated inhibitory signaling [49].

In summary, the co-inhibitory pathway in the B7-CD28 family provides a key inhibitory signal that regulates immune homeostasis and defense and protects tissue integrity. These co-inhibitory signals limit the intensity and duration of the immune response, thereby inhibiting immune-mediated tissue damage, regulating the inflammatory response, and organizing the occurrence of autoimmunity. The B7-CD28 family pathway can control the initial activation of primary T cells, regulate the differentiation and function of effector cells, memory cells, and regulatory T cells, and maintain immune homeostasis and immune tolerance.

## 5 Treatment of autoimmune uveitis

### 5.1 Immunomodulatory targets and drugs for the treatment of autoimmune uveitis

In recent years, the application of immunomodulatory targets to treat autoimmune uveitis and autoimmune diseases has attracted considerable attention. The main goals of these methods are to control acute immune and inflammatory responses and inhibit chronic responses [50]. Experimental research and clinical trials by blocking the effector pathway, or blocking its accompanying co-stimulatory molecules at different checkpoints of the immune response, such as T-cell receptor (CD3) and its co-stimulatory receptors CD28, CTLA-4, and corresponding ligands (CD80 and CD86), have achieved good results in the treatment of autoimmune uveitis [51,52,53]. Inhibition of immune responses via blockade of the CD28 receptor B7(CD80 and CD86) remains a valid option for autoimmune diseases and is being explored through the use of Abatacept (CTLA-4-Ig), a second-generation recombinant fusion protein consisting of CTLA-4 and a modified Fc fragment of human IgG1 that binds to both CD80 and CD86. Abatacept is approved by the Food and Drug Administration (FDA) for the treatment of rheumatoid arthritis and juvenile idiopathic arthritis and shows promising results in the case of systemic lupus erythematosus patients. For the treatment of uveitis, only adalimumab has received FDA approval [54]. However, biologics that target T-cell receptors or effector functions are also becoming more and more popular. Examples include blocking T-cell signaling pathways (with cyclosporine, FK-506, and rapamycin); inhibiting CD4 T-cell function (with anti-IL-2R and anti-IFN-γ Abs); focusing on TNF-α (with etanercept, infliximab, and thalidomide); and developing biologicals that target immune modulatory motors (adhesion, co-stimulatory motors) [55]. Abe developed the anti-CD28 monoclonal antibody plasmalemmal vesicle-associated protein (PV1), which is an anti-CD28 monoclonal antibody. The study revealed that PV1 effectively reduces the inflammatory response and the incidence of uveitis. It achieves this by partially suppressing the activity of effector T cells. Notably, PV1 specifically decreases the population of Th1 cells, while its impact on Th17 cells is not significant. Additionally, PV1 does not influence the number of Th17 cells or the production of IL-2. It is worth noting that Th17 cells also contribute to the progression of autoimmune uveitis [56,57], highlighting that PV1 still holds significant research potential in the treatment of this condition. Moreover, cell therapy using tolerogenic DCs (tolDC) for autoimmune diseases is currently a hot topic of research. However, one of the major obstacles in this area is the production of stable tolDC. Recent studies have shown that some researchers have discovered that a single subcutaneous injection of 2-dtoldc in mice can prevent experimental autoimmune uveitis [57,58]. Although further research is needed to determine its applicability in humans, this method shows promise as an effective treatment approach for autoimmune uveitis.

### 5.2 The treatment goals of autoimmune uveitis

The treatment goals of autoimmune uveitis are to control inflammation, prevent recurrence, protect eyesight, and reduce adverse drug reactions [58]. The current standard of treatment for autoimmune uveitis typically involves the use of corticosteroids as the first-line drugs. However, in some cases, additional immunosuppressive agents may be necessary. For patients who are intolerant or unresponsive to conventional immunosuppressive therapy, biological agents have emerged as an effective treatment option. Over the past 20 years, the introduction of biological agents has significantly influenced the clinical management of autoimmune uveitis. The latest uveitis treatment guidelines recommend a step therapy approach, wherein the use of different treatment options is sequentially considered based on the response to previous treatments. Furthermore, targeted therapy, which involves blocking specific immune targets, has become a prominent area of research in recent years. However, due to the high cost associated with these agents, further investigation is needed to explore their efficacy, safety, and long-term outcomes. Nonetheless, targeted therapy holds promise for improving the treatment of autoimmune uveitis.

## 6 Conclusion

NIU is the most prevalent type of uveitis, accounting for approximately 55% of cases in China. It is an autoimmune disease that affects multiple organs. EAU serves as an animal model for studying NIU in humans. EAU is induced in susceptible animals through the activation of T cells by retinal antigens. This model is widely employed to investigate the etiology, mechanisms, and prevention of uveitis in humans. As obtaining clinical specimens of uveitis raises ethical concerns, current research heavily relies on EAU animal models. However, it is important to acknowledge that the evidence derived from animal models has limitations in evaluating immune function, thus warranting cautious interpretation.

Autoimmune uveitis is an autoimmune disease mediated by Th cells, and the imbalance in the number and proportion of Th-cell subsets is an important mechanism for the pathogenesis of uveitis. DCs play a crucial role in regulating the proportion of various cell subpopulations in uveitis and are a central link in initiating, regulating, and maintaining immune responses. At present, there are still some unsolved issues, regarding autoimmune uveitis, such as unclear diagnosis, difficulty to clarify the cause, and differentiation of treatment options.

This review provides an overview of DC-induced uveitis, including its characteristics and current status. It discusses factors that can influence the status of DCs and explores potential treatment methods for targeting DCs in both experimental autoimmune uveitis animal models and humans.

Furthermore, the review emphasizes the importance of researching molecular pathways and signaling pathways that regulate DC function. For example, investigating molecules such as CTLA-4, which inhibits the B7-CD28 co-stimulatory interaction, can enhance immune homeostasis. The aim is to identify new therapeutic targets and develop targeted strategies for DCs, such as DC vaccine therapy or the use of immune modulators. These approaches can help regulate abnormal immune system activation and inflammatory responses.

The review also highlights the significance of developing personalized treatment strategies based on the immune characteristics and disease manifestations of individual patients. This includes the personalized design of DC therapy and precise medication, aiming to improve treatment effectiveness while minimizing adverse reactions.

In summary, DCs and their associated pathways hold great potential for innovation and application in the study of NIU. They offer new ideas and methods for treating this disease. However, further comprehensive research, both in basic and clinical settings, is necessary to validate their efficacy and safety, enabling their translation into clinical applications.
